# To what degree do patients actively choose their healthcare provider at the point of referral by their GP? A video observation study

**DOI:** 10.1186/s12875-019-1060-2

**Published:** 2019-12-01

**Authors:** Amy J. C. Potappel, Maartje C. Meijers, Corelien Kloek, Aafke Victoor, Janneke Noordman, Tim olde Hartman, Sandra van Dulmen, Judith D. de Jong

**Affiliations:** 1 Nivel (Netherlands institute for health services research), Utrecht, Netherlands; 2 Research Group Innovation of Human Movement Care, HU University of Applied Sciences Utrecht, Utrecht, Netherlands; 3 Radboud University Medical Center, Radboud Institute for Health Sciences, Department of Primary and Community Care, Nijmegen, the Netherlands; 4 Donders Institute for Brain Cognition and Behaviour, Radboudumc Nijmegen, Nijmegen, Netherlands; 5 Faculty of Health and Social Sciences, University of South-Eastern Norway, Drammen, Norway; 6 Maastricht University, Department of Health Services Research, Faculty of Health, Medicine and Life Sciences, Maastricht, Netherlands

**Keywords:** Patient choice, Healthcare providers, Referral, Healthcare reform, Physicians’ role, General practitioners, Health insurer, Health insurance, Communication

## Abstract

**Background:**

Many countries in Europe have implemented managed competition and patient choice during the last decade. With the introduction of managed competition, health insurers also became an important stakeholder. They purchase services on behalf of their customers and are allowed to contract healthcare providers selectively. It has, therefore, become increasingly important to take one’s insurance into account when choosing a provider. There is little evidence that patients make active choices in the way that policymakers assume they do. This research aims to investigate, firstly, the role of patients in choosing a healthcare provider at the point of referral, then the role of the GP and, finally, the influence of the health insurer/insurance policies within this process.

**Methods:**

We videotaped a series of everyday consultations between Dutch GPs and their patients during 2015 and 2016. In 117 of these consultations, with 28 GPs, the patient was referred to another healthcare provider. These consultations were coded by three observers using an observation protocol which assessed the role of the patient, GP, and the influence of the health insurer during the referral.

**Results:**

Patients were divided into three groups: patients with little or no input, patients with some input, and those with a lot of input. Just over half of the patients (56%) seemed to have some, or a lot of, input into the choice of a healthcare provider at the point of referral by their GP. In addition, in almost half of the consultations (47%), GPs inquired about their patients’ preferences regarding a healthcare provider. Topics regarding the health insurance or insurance policy of a patient were rarely (14%) discussed at the point of referral.

**Conclusions:**

Just over half of the patients appear to have some, or a lot of, input into their choice of a healthcare provider at the point of referral by their GP. However, the remainder of the patients had little or no input. If more patient choice continues to be an important aim for policy makers, patients should be encouraged to actively choose the healthcare provider who best fits their needs and preferences.

## Background

**Fig. 1:**
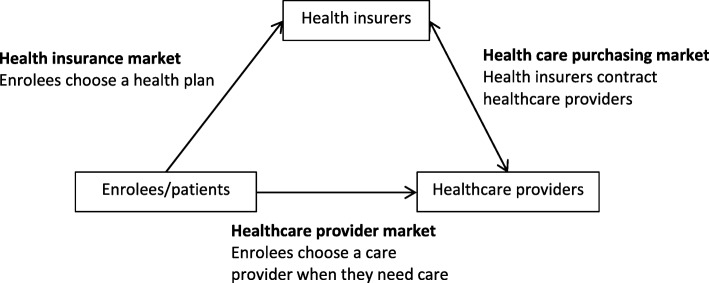
Model of healthcare market in a system of managed competition

During the last decade, in many European countries healthcare systems are reformed towards a demand-oriented care system in order to improve the quality of care and to contain costs [1–3]. Central regulation of the provision of health care is replaced by a system based on flexible markets in which consumers can express their demands and in which the providers can meet these demands [4]. In several countries, for example, Switzerland, Belgium, Germany, and the Netherlands, these reforms were based on introducing managed competition with more patient choice of healthcare provider and insurance policy [5, 6]. Within these healthcare systems health insurers were given an important role to ensure the public interest of quality, accessibility and affordability of care. The intention was to create a competitive healthcare system in which three players interact on three healthcare markets: health insurers compete for enrollees/patients on the health insurance market and healthcare providers compete for patients/enrollees on the healthcare provision market. On the healthcare purchasing market, health insurers negotiate with care providers (Fig. 1) [6, 7].

People were given a free choice of a health insurer and the possibility of switching health insurer annually on the health insurance market. For a well-functioning market, people must be able to observe and experience differences in health insurance policies. Only when they have the right information and can compare policies, they could keep health insurers sharp on the price and quality of the policies they offer [8]. The possibility that people could switch, was intended to lead to competition between health insurers which, in turn, would enhance allocative and productive efficiency, since they also bear financial risk. The possibility that enrollees could switch aimed to encourage responsiveness to their preferences among health insurers. Health insurers would, as a consequence, be encouraged to purchase services more critically from healthcare providers on the healthcare purchasing market, while paying attention to the price and quality. If patients are more sensitive to the price and quality, then insurers would try to maintain or improve the quality while, at the same time, ensuring that healthcare remains affordable [5, 9].

A free patient choice of healthcare provider on the healthcare provider market was also introduced, in order to increase the efficiency of healthcare. It was assumed that by letting patients choose the qualitatively best or most effective healthcare provider, this would then send signals to healthcare providers who perform poorly. This should increase the competition between healthcare providers to improve and maintain their quality. As a result, healthcare providers would, in turn, offer more demand-driven care, which would better fit the situation of the individual patient [6, 9].

Although patient choice is important in healthcare systems based on managed competition, their choice may seem limited since insurers are allowed to contract healthcare providers selectively. Depending on the insurance policy patients opted for, they may be required to pay part of their healthcare themselves when visiting a provider who has no contract with their health insurer. If health insurers are transparent about the insurance products they offer, the providers they contracted and the quality and costs of treatment, patients would be enabled to make trade-offs, depending on their needs and preferences, between different healthcare providers. Policymakers assume that patients behave as rational healthcare consumers and actively choose the provider that best fits their needs and preferences based on this information and comparative information that is provided by other parties such as the government [10]. However, there seems to be little evidence that patients actually use this information [11, 12]. Instead, it seems that the recommendation of patients’ general practitioner (GP) plays a major role in patients’ choices of providers [9, 13–15].

In one third of European countries GPs function as gatekeepers regulating access to specialist care [16]. Doctors are required to treat individual patients to the best of their ability and, simultaneously, they are expected to fulfil a duty to society to make the most equitable use of resources overall [17]. Because patients often rely on their GP to decide on the course of action that is to be taken following the consultation, a patient’s GP might be the right person to provide them with information about treatment alternatives, ideally including other healthcare providers they could choose [18]. GPs might point out to their patients that costs tend to differ between healthcare providers, encourage them to use the available choice information, and involve them in making a decision about a healthcare provider [14, 19, 20]. However, when referring, GPs seem to rely more on informal sources such as feedback from colleagues and patients, as well as on their own experience of cooperating with a hospital or department [19]. The distance to a hospital was the factor most often used by GPs when choosing a hospital on behalf of their patients [19]. After all, GPs also have insufficient information about providers and the length of a consultation may be too limited to be able to look up and discuss choice information [21].

There seems to be little evidence that patients actively choose a healthcare provider themselves [22–25] and actually use the available choice information [11, 12]. However, since the implementation of managed competition and patient choice, much has been done by the government and health insurers to get patients more actively involved in their own healthcare, for instance by making comparative information more accessible and available to them. For example, several websites were developed to enable patients to compare healthcare providers (e.g. www.kiesbeter.nl). This warranted a repetition of earlier research. Additionally, unlike most other studies, we observed if the topic of health insurer or insurance was discussed during consultations, given the role that the insurer is now expected to play in the Dutch healthcare system.

### Research aim

By examining everyday consultations between GPs and their patients, we aim to investigate if, and how, patient choice is reflected at the point of referral (i.e. how policy works in practice). That is to say do GPs inform patients about different options and do patients and their GPs take the patients’ health insurance policy into account. We answer the following research questions: 1) What role does the patient play in choosing a healthcare provider at the moment of referral by their GP?, 2) At the moment of referral, what role does the GP play in their patients’ choices of a healthcare provider?, and 3) What is the influence of the patient’s health insurer or insurance policy in the choice of a healthcare provider at the point of referral? With ‘consultation’ we mean the process of getting advice from a GP and we define ‘referral’ as the transfer of care for a patient from the GP to another doctor or clinic for further diagnosis and/or treatment.

## Method

### Recruitment of professionals

The video recordings were collected as part of a study that aimed to investigate GP-patient communication [26]. In 2015, 36 GPs from the eastern part of the Netherlands were approached. In 2016, a further 44 GPs, located in other parts of the country, were approached. The GPs were approached by the researchers via their network and through participation in earlier studies from Nivel (Netherlands institute for health services research) and the Radboudumc (Radboud university medical center). There were no inclusion, or exclusion, criteria for GPs to participate. GPs and patients were told that the study was about GP-patient communication but were unaware that their referral decisions were being analysed.

### Recruitment of patients and procedure

The 28 GPs agreed to videotape consecutive, standard consultations on one or two random days. The recordings were carried out with an unmanned digital camera. In 2015, 508 patients were approached. Another 162 patients were approached in 2016. All GPs and patients who participated signed an informed consent form before the recording of the consultation. Participants could withdraw their consent at any time, but none of them did. Prior to the consultation, patients completed a questionnaire about, among other things, their sociodemographic characteristics and their expectations of the consultation. Patients younger than 18 years of age, and patients who did not speak Dutch adequately, were excluded. GPs filled in a registration form after every contact with a patient, assessing some clinical aspects, plus a one-off questionnaire about their background characteristics [26].

### Analyses

Three researchers (AP, MM and CK) observed each one third of the video-recorded consultations. An observation protocol was developed based on an already existing protocol from the 2007/2008 study [25]. Additional questions were formulated about the health insurer or insurance. First, five consultations were observed by the three observers together to test the observation protocol and to make adjustments. For example, a response category ‘not applicable’ was added for some questions. Additionally, each time, after about twenty-five recordings, the observers discussed difficulties they encountered and to reach consensus about how to fill out the protocol.

When more than one medical condition was discussed during a consultation, the first one mentioned was used for filling in the observation protocol. The questions of the observation protocol as used for this study consisted of 14 items (see Additional file 1), which addressed the following topics:

1. The referral in general. This comprised three items, i.e. the kind of healthcare provider – such as a specialist or a physiotherapist - the patient was referred to during the consultation, the specific type of provider the patient was referred to, and the reason for referral.
2. The role of the patient in choosing a healthcare provider was observed using four items. These included how much input patients had in the choice of healthcare provider (see Table 1 for the question from the observation protocol that assessed how much input patients had), if they preferred a certain healthcare provider, who mentioned the provider alternative(s) first, and the moment at which the patients pronounced their preferences.
3. The role of the GP in the patient’s choice of a healthcare provider was observed using five items. These items included whether the GP asked for the preference of the patient for a healthcare provider, discussed multiple provider alternatives, had a preference, why the GP opted for a particular provider and if the GP provided information about providers.
4. The influence of the health insurer, or the insurance policy of the patient in the choice of a healthcare provider was observed using two items: if topics regarding the health insurance of the patient, such as the reimbursement of a specialist, were discussed, and who took the initiative to discuss these topics.

A random 10 % of the consultations were rated by three observers (AP, MM and CK) independently in order to assess interrater reliability. It resulted in a Kappa score of 0.64 (range 0.45–0.92), indicating substantial reliability [27].

**Table 1:** How much input is there from the patient around the choice of a healthcare provider?

How much input is there from the patient around the choice of a healthcare provider?		
□ 1) little or no input	□ 2) some input	□ 3) a large amount of input
1 = little or no input. The GP chooses the provider and the patient simply agrees with the proposed institution or caregiver. It is obvious that the patient follows up the advice of the GP.		
2 = some input. The patient is given a choice by the GP between a few providers or tells the GP that he or she does not want to be referred to a specific provider.		
3 = a large amount of input. Not the GP, but the patient him or herself chooses the provider he or she is referred to or asked for alternative options. Alternatively, no decision is made during the consultation and the patient has to choose a care provider after the consultation.		

### Statistical analyses

STATA 15 was used for performing the descriptive analyses and the interrater reliability calculation. Neither determining causation nor performing statistical analyses were attempted due to the explorative and qualitative nature of the data.

### Ethical considerations

The study was carried out according to Dutch privacy legislation. The privacy regulations were approved by The Dutch Data Protection Authority. Approval by a medical ethics committee was not required under Dutch law for this observational study. Written informed consent was obtained from all GPs and patients, prior to consultation. All participants could withdraw their consent at any time, however, as previously mentioned, none of them did.

## Results

In 2015, 20 GPs agreed to participate (56% participation rate) and in 2016, eight GPs participated (18% response). The number of videotaped consultations per GP ranged from 1 to 29. In 2015, 392 patients (77% participation rate) participated and another 102 (63% participation rate) in 2016. In total, 475 consultations were recorded (recording failed of 19 consultations). For the current analyses, 117 consultations (25%) were used in which a referral to a healthcare provider took place. In the other consultations, the GP prescribed medication or a medical aid, but no referral took place.

### Patient and GP characteristics

The 117 patients in this study were referred to a variety of healthcare providers, such as mental healthcare providers or other medical specialists such as gynecologists or dermatologists. The three types of healthcare providers that people are most referred to, were radiologists for x-rays (*n* = 14), physiotherapists (*n* = 12), and to ear, nose and throat (ENT) doctors (*n* = 10). A smaller number of people were, for example, referred to psychologists (*n* = 5), neurologists (n = 5), dermatologists (n = 5), ophthalmologists (*n* = 3) or orthopedics (n = 3). There were also specific types of healthcare providers for which only one patient was referred to, such as to providers practicing haptonomy (a treatment in which touch between the therapist and the patient can contact with the feelings that are stored in the body).

Table 2 describes the characteristics of the patients and the GPs involved in the 117 consultations in which a referral to a healthcare provider took place. The majority of both GPs and patients was female. Patients were, on average, 52.1, and GPs, 47.7 years of age. Most patients had a medium educational level.

**Table 2:** Background characteristics of the patients and the GPs per patient group

	Total (*N* = 117)	No/little input (*N* = 51)	Some input (*N* = 29)	A lot of input (*N* = 37)
GP (*N* = 28)				
Age in years (M (SD))	47.7 (10.1)	47.8 (10.9)	48.6 (12.6)	46.0 (9.3)
Gender (n(%))				
Male	12 (42.9)	9 (40.9)	8 (57.1)	6 (30.0)
Female	16 (57.1)	13 (59.1)	6 (42.9)	14 (70.0)
Patient				
Age in years (M (SD))^1^	52.1 (16.9)	51.8 (17.3)^1^	51.1 (14.9)	53.2 (18.1)
Gender (*n*(%))				
Male	49 (41.9)	19 (37.3)	12 (41.4)	18 (48.7)
Female	68 (58.1)	32 (62.8)	17 (58.6)	19 (51.4)
Educational level (n(%))				
None	4 (3.4)	0 (0.0)	3 (10.3)	1 (2.7)
Low^2^	7 (6.0)	4 (7.8)	2 (6.9)	1 (2.7)
Medium^3^	65 (55.6)	29 (56.9)	13 (44.8)	23 (62.2)
High^4^	39 (33.3)	17 (33.3)	11 (37.9)	11 (29.7)
Missing	2 (1.7)	1 (2.0)	0 (0.0)	1 (2.7)
Reason for referral (n(%))				
Diagnosis	67 (100.0)	32 (47.8)	22 (32.8)	13 (19.4)
Treatment	65 (100.0)	28 (43.1)	11 (16.9)	26 (40.0)
Second opinion	1 (100.0)	0 (0.0)	0 (0.0)	1 (100.0)

^1^The age was missing for 1 patient; ^2^Low = primary school; ^3^Medium = secondary school or intermediate vocational training; ^4^High = high vocational education

### Role of the patient

Patients differed in the amount of input on the referral decision at the point of referral. Based on item 4 in the observation protocol (i.e. how much input is there from the patient around the choice of a healthcare provider?), the patients were divided into three groups. The first group had little or no input in the choice of a healthcare provider (*n* = 51(44%)). The GPs chose the healthcare providers and patients simply agreed with the proposed option. It is obvious that the patient follows the advice from the GP. For example, the GP would say: “You have a referral letter and you have to call this number to make an appointment”. The GP did not even mention the name of the hospital the patient was being referred to.

The second group of patients had a large amount of input into the choice of a healthcare provider (*n* = 37(32%)). These patients chose, themselves, the healthcare provider they were referred to, or asked for alternative options. For example, a patient said: “I want to go to this specialist, because my mother has also been there for the same problem”.

The third group falls between the first and second group. This group consisted of patients who were either given a choice of several options by their GP, or told their GP that they did not want to be referred to a specific provider (*n* = 29(25%)). For example, the GP would ask: “Do you prefer to go to hospital A or to hospital B?”

The groups differ regarding expressing a preference for a certain healthcare provider and who mentioned the referral destination options first: the GP or the patient (Table 3).

**Table 3:** The role of the patient per amount of input group

	No/little input (n(%))	Some input (n(%))	A lot of input (n(%))
	51 (44%)	29 (25%)	37 (32%)
Does the patient prefer a particular healthcare provider?			
Yes	6 (11.8%)	23 (79.3%)	35 (94.6%)
No	45 (88.2%)	6 (20.7%)	2 (5.4%)
Who mentions the option(s) in terms of a healthcare provider as first?			
GP	35 (68.6%)	26 (89.7%)	8 (21.6%)
Patient	3 (5.9%)	3 (10.3%)	27 (73.0%)
Unclear	13 (25.5%)	0 (0.0%)	2 (5.4%)

### Role of the GP

In 47% (*n* = 55) of the consultations, the GP asked the patient about their preference for a healthcare provider. For instance, this could be by asking: ‘Do you want to go to hospital A or hospital B?’ In 36% (*n* = 42) of the consultations, the GP discussed multiple options regarding healthcare providers. In more than half of the consultations (63% (*n* = 74)) the GP, him or herself, preferred a particular healthcare provider. And in 73% (*n* = 54) of those consultations, the GPs gave this preference on their own initiative and not at the request of the patient. It was often not known why the GP was referring the patient to a certain healthcare provider. In about half of the consultations (55% (*n* = 64)), the GP did provide the patients with extra information about the healthcare provider they referred the patient to. This mostly concerned practical information, such as opening times and the location of the provider (72% (*n* = 46)). In six consultations, extra information was given about the quality or the costs of a particular healthcare provider.

### Influence of the health insurer or insurance policy

Topics regarding the health insurance of the patient were discussed in sixteen out of the 117 consultations (14%). Table 4 shows which topics were discussed and how many times they were mentioned as a percentage of the total of 117 consultations. The reimbursement of a treatment by a specialist was the topic that was discussed most often. This was followed by the deductible for the patient. For instance, the GP said: “If you allow yourself to be sterilised by the GP instead of the hospital, you do not have to pay a deductible”. Another example was a patient who said: “You can prescribe the more expensive variant of the asthma puffer, because I have already paid my entire deductible”. The supplementary health insurance of the patient was discussed in three of the consultations. For instance, the GP asked: “Do you have physiotherapy in your supplementary insurance package?” Five topics from the observation protocol regarding the health insurance of the patient were never discussed during the 117 consultations in which a referral for a healthcare provider took place. These, for example, included the reimbursement of a medicine. In more than half of the consultations in which the health insurance was discussed (54%(*n* = 7)) the GP took the initiative.

**Table 4:** Topics discussed regarding the health insurance of the patient on referral to a healthcare provider

Topics discussed regarding the health insurance	*N* (%^1^)
Reimbursement of a treatment by a specialist	6 (5.1%)
The deductible of the patient	4 (3.4%)
The supplementary health insurance of the patient	3 (2.6%)
Reimbursement of a treatment by a GP practice nurse in mental health	3 (2.6%)
Reimbursement of a treatment by a paramedic	3 (2.6%)
Other topic regarding the health insurance	3 (2.6%)
The current health insurance/insurer of the patient	0 (0.0%)
Reimbursement of a medicine	0 (0.0%)
Reimbursement of specific tools	0 (0.0%)
Waiting list mediation	0 (0.0%)
Help from a health insurer in choosing a healthcare provider or with comparing different healthcare providers	0 (0.0%)

^1^The percentage as a total of the 117 consultations in which a referral to a healthcare provider took place

## Discussion

Over half of the patients from this study (*n* = 117 (57%)) had some, or a lot of, input into the choice of a healthcare provider at the point of referral. The other 43% of the patients had little or no input, meaning that the GP chose a healthcare provider for them. Differences were visible in how patient choice is incorporated during the consultation. The influence of the patient’s health insurer or insurance policy in choosing a healthcare provider at the point of referral is minimal, insofar as this decision is taken within the GP consultation. In only 14% of the consultations topics regarding the health insurer or insurance policy of the patient were discussed.

### Comparison with existing literature

In 2015/2016, more patients seem to have some, or a lot of, input into the decision to choose a healthcare provider compared to 2007/2008 [25]. However, we still found that in most consultations the GP chose the hospital or specialist on behalf of the patient. Existing literature has already pointed out that patients usually visit the healthcare provider that is recommended by their GP [9, 13–15, 19, 21, 28, 29]. GPs themselves also experience that the patients’ demand for choice during their referral is limited and usually expressed in a demand to be sent to the nearest hospital [30].

Patients do not seem to act as actively as presumed by policymakers, which firstly might be due to the fact that the gatekeeping function performed by primary care has a strong foundation in the Netherlands [30]. This means that the GP influences the referrals to specialist care, which might result in patients feeling as if they do not have the autonomy to make their own decisions. However, it might also be that the quality and the duration of the relationship between the GP and the patient influences the form decision-making takes. In a long-term continuous relationship with the patient GPs often are familiar with the preferences of the patient and act on them [17]. Secondly, the duration of consultations in the Netherlands is only ten minutes, which might be too short to discuss multiple referral options. As a result, GPs might opt for the obvious option. Thirdly, patients might experience a lack of insight into the quality of healthcare providers and are, therefore, reluctant to decide on a healthcare provider themselves [31]. Lastly, patients may not feel that the choice of a provider is as important as policymakers do [32].

Although GPs are divided about if discussing costs issues with patients belongs to the profession’s job responsibilities, all providers are obliged to provide patients with the information that is relevant for them to be able to make an informed choice [33, 34]. Besides, GPs are expected to involve their patients into decisions about their care and to aid to empowering them to do so [20, 35, 36]. However, it is demonstrated in this study that most GPs do not involve patients in the decision-making process regarding a referral destination during consultation. Studies indicate that, because of a lack of time, the GP cannot reveal all information about the different possibilities when advising patients about their choice [6, 30]. GPs refer patients to a particular hospital, for instance because their care history is known there or their diagnosis is unknown at the moment of referral because of which they are unable to refer to a hospital that specializes in their condition [24]. This was also demonstrated in the results of this study. In most of the consultations other referral options were not discussed and only practical information about the healthcare provider seems to have been provided to patients. The reason for referring to a specific healthcare provider was rarely explained to the patient.

Lastly, our results indicate that topics regarding the health insurer/insurance policy of the patient are barely (*N* = 16 (14%)) taken into account when choosing a healthcare provider. This aligns with previous research that found that of 219 people, only 22 (10%) said that their health insurer played a role in their decision to choose a hospital or specialist [37]. A possible explanation for this might be that it is complicated and time-consuming for GPs to discuss topics regarding the health insurance of a patient because each patient has a different insurer and is insured differently. Policymakers and health insurers might expect GPs to inform patients about the consequences that their referral decisions can have. However, it could also be that GPs and patients do not see the point of discussing matters about costs instead of medical content during their consultation time. Health insurers themselves should inform GPs and patients of the importance of discussing matters around the health insurance policy of the patient. They should also provide patients with information about healthcare providers to enable them to make choices. A last reason that the health insurance of the patient is seldom taken into account during referral might be that, in the Netherlands, the consequences, especially financial ones of selective contracting are barely visible. Nowadays, selective contracting rarely occurs and patients are nonetheless compensated for most costs incurred at non-contracted providers, but selective contracting is expected to gain more importance over the next few years [38]. Therefore, it will become more important for patients, and for GPs if they want to support patient decision making, to take patients’ health insurance into consideration when choosing a healthcare provider.

### Strengths, limitations and further research

Few studies have analysed actual GP-patient consultations in order to study the patients’ role in the referral decision, and GPs’ support for patients who are actively choosing a provider. Observations are a more objective source of information than self-reporting by patients or GPs, which could be biased. In addition to the first study from 2007 to 2008 [25], this study has also observed the role that the health insurer or insurance policy of the patient plays in making a decision on a healthcare provider at the point of referral. A final strength of this study is that the GPs participating were unaware of the fact that the observations focused on referral decisions. Therefore, the Hawthorne effect, a possible limitation of observational research, is minimal and our results mirror the actual daily situation in general practice.

A limitation of this study is that observations do not give insight into the underlying motives for behaviour and attitudes. For example, what is the underlying reason for a GP to send someone to a specific hospital? It is also unclear if patients complied with their GP’s advice or went anywhere else. Neither was any account taken of the behaviour of the same patients in previous consultations or with the possible existing doctor-patient relationship that could influence the input of a patient about the choice of a healthcare provider. In addition, we did not look at whether patients or GPs find it relevant to discuss topics regarding the health insurance of the patient. Another limitation is that 20 of the 28 GPs were from the east of the Netherlands, because of which our results might not be generalizable to an entire population. Nevertheless, our sample matches the population of Dutch GPs with regard to age and gender [39]. Yet another limitation is that the lowest Kappa score that we had (0.45), was moderate [27]. However, the agreement score for this item was better (mean 64.9%). Nevertheless, our results should be considered with some caution. Lastly, there were differences in how many patients were observed per GP, ranging from one patient to 29 patients. However, conducting multilevel analysis was unnecessary, because the observations per question of the observation protocol for each GP were distributed, meaning that, for instance, a specific GP did not always ask patients for their preferences, but sometimes did while, at other times, did not.

Future research could focus on the importance of costs in making a decision to choose a healthcare provider during a GP consultation. Thus, investigating whether the idea of the importance of taking the health insurer or insurance policy into account when choosing a particular healthcare provider is something which is gaining support from both the GP and the patient. Further research could also focus on the importance of shared decision-making when choosing a healthcare provider during GP consultations. This means investigating what GPs and patients think of the idea that GPs are expected to encourage patients to make an active choice about a healthcare provider. Furthermore, it is interesting to study if an active choice makes a difference for health outcomes.

## Conclusion

If managed competition with active patient choice is to work as intended by policy makers, it is important that patients make active choices of healthcare providers. Ideally, they should consider the quality and out-of-pocket costs of the different options, the latter depending on their health insurance policy. Our results seem to indicate that the policy regarding the implementation of patient choice is still only partially reflected in daily practice. The expectations, arising from policy, for patients and GPs in choosing a healthcare provider may be unrealistic. Discussing referral options is time consuming and GPs, just like patients, lack quality information and do not have insight into patients’ insurance policies. Besides, the consequences for patients of selective contracting are currently barely visible for them in the Dutch healthcare system, as it does not yet take place very often. With the role that health insurers have within the current Dutch healthcare system, they can be expected to distribute more information about healthcare providers and selective contracting among patients and GPs. Decision aids could also help patients to choose a particular healthcare provider during the consultation and GPs in supporting their patients in making active choices. Patients could, in turn, indicate their preferences regarding providers and making active choices, and what they expect from their GP more clearly to their GP. Some patients might not be interested in playing an active part in choosing a healthcare provider, or are not capable of doing so, and would rather delegate this task to their GP, while others do want to play an active role in their care choices.

## Supplementary information

**Additional file 1.** Observation protocol.

## Data Availability

The datasets used and/or analysed during the current study are available from the corresponding author on reasonable request.

## Abbreviations

GP: General practitioner

## References

[1] Laske-Aldershof T, Schut E, Beck K, Greß S, Shmueli A, van de Voorde C (2004). Consumer mobility in social health insurance markets. Appl Health Econ Health Policy.

[2] Saltman RB, Figueras J (1998). Analyzing the evidence on European Health care reforms: experience in western European health care systems suggests lessons for reform in the United States, according to a major international comparison by the World Health Organization. Health Aff (Millwood).

[3] van de Ven WP, Beck K, Buchner F, Schokkaert E, Schut FE, Shmueli A, Wasem J (2013). Preconditions for efficiency and affordability in competitive healthcare markets: are they fulfilled in Belgium, Germany, Israel, the Netherlands and Switzerland?. Health Policy.

[4] Van der Kraan WGM, Van der Grinten TED (2004). The development of Demand-driven care as a new governance concept. NIG Annual Work Conference 2004.

[5] Thomson S, Busse R, Crivelli L, van de Ven W, van de Voorde C (2013). Statutory health insurance competition in Europe: a four-country comparison. Health Policy.

[6] Vrangbaek K, Robertson R, Winblad U, van de Bovenkamp H, Dixon A (2012). Choice policies in northern European health systems. Health Econ Policy Law.

[7] Kroneman M, Boerma W, van den Berg M, Groenewegen P, de Jong J, van Ginneken E (2016). The Netherlands: health system review. Health Syst Transit.

[8] Klop K, Ed J (2002). Marktwerking in de gezondheidszorg. Markt en weaarden.

[9] Reitsma M, Brabers A, Masman W, de Jong J (2012). De kiezende burger.

[10] Groenewoud S, Van Exel NJA, Bobinac A, Berg M, Huijsman R, Stolk EA (2015). What influences patients' decisions when choosing a health care provider? Measuring preferences of patients with knee arthrosis, chronic depression, or Alzheimer's disease, using discrete choice experiments. Health Serv Res.

[11] Hibbard JH, Peters E, Health ARP (2003). Supporting informed consumer health care decisions: data presentation approaches that facilitate the use of information in choice. Annu Rev Publ Health.

[12] Trisolini MG, Isenberg KL (2007). Public reporting of patients survival (mortality) data on the dialysis facility compare web site. Dial Transplant.

[13] Dealey C (2005). The factors that influence patients’ choice of hospital and treatment. Br J Nurs.

[14] Magee H, Davis LJ, Coulter A (2003). Public views on healthcare performance indicators and patient choice. J R Soc Med.

[15] Merle V, Germain JM, Tavolacci MP, Brocard C, Chefson C, Cyvoct C, Edouard S, Guet L, Martin E, Czernichow P (2009). Influence of infection control report cards on patients’ choice of hospital: pilot survey. J Hosp Infect.

[16] Group; LMPPP (2018). Primary Care in Europe Compared.

[17] Jones IR, Berney L, Kelly M, Doyal L, Griffiths C, Feder G, Curtis S (2004). Is patient involvement possible when decisions involve scarce resources? A qualitative study of decision-making in primary care. Soc Sci Med.

[18] Langer A, Schröder-Bäck P, Brink A, Eurich J (2009). The agency problem and medical acting: an example of applying economic theory to medical ethics. Med Health Care Philos.

[19] Birk HO, Henriksen LO. Which factors decided general practitioners’ choice of hospital on behalf of their patients in an area with free choice of public hospital? A questionnaire study. BMC Health Serv Res. 2012;12(1). 10.1186/1472-6963-12-126 PMC3407516 22630354

[20] Winblad U (2008). Do physicians care about patient choice?. Soc Sci Med.

[21] Rosen R, Florin D, Hutt R (2007). An anatomy of GP referral decisions. A qualitative study of GPs’ views on their role in supporting patient choice.

[22] Harris KM (2003). How do patients choose physicians? Evidence from a national survey of enrollees in employment-related health plans. Health Serv Res.

[23] Stacey D, Légaré F, Lewis K, Barry M, Bennett C, Eden K, Holmes-Rovner M, Llewellyn-Thomas H, Lyddiatt A, Thomson R, et al. Decision aids for people facing health treatment or screening decisions. Cochrane Database Syst Rev. 2017;4. 10.1002/14651858.CD001431.pub5 PMC6478132 28402085

[24] Victoor A (2015). (How) do patients choose a healthcare provider?.

[25] Victoor A, Noordman J, Sonderkamp JA, Delnoij DM, Friele RD, van Dulmen S, Rademakers JJ. Are patients’ preferences regarding the place of treatment heard and addressed at the point of referral: an exploratory study based on observations of GP-patient consultations. BMC Fam Pract. 2013;**14**(1). 10.1186/1471-2296-14-189 PMC4029442 24325155

[26] Houwen J, Lucassen PL, Stappers HW, Assendelft PJ, van Dulmen S (2017). Medically unexplained symptoms: the person, the symptoms and the dialogue. Fam Pract.

[27] Sim J, Wright CC. The kappa statistic in reliability studies: use, interpretation, and sample size requirements. Phys Ther. 2005;8(3). 15733050

[28] Dijs-Elsinga J, Otten W, Versluijs MM, Smeets HJ, Kievit J, Vree R, Marang-van de Mheen PJ (2010). Choosing a hospital for surgery: the importance of information on quality of care. Med Decis Mak.

[29] Rademakers J, Nijman J, Brabers AEM, de Jong JD, Hendriks M (2014). The relative effect of health literacy and patient activation on provider choice in the Netherlands. Health Policy.

[30] Dixon A, Robertson R, Bal R (2010). The experience of implementing choice at point of referral: a comparison of the Netherlands and England. Health Econ Policy Law.

[31] Damman OC, Rademakers J (2008). Keuze-informatie in de zorg: een internationale vergelijking van presentatiewijzen op internet.

[32] Fotaki M, Roland M, Boyd A, McDonald R, Scheaff R, Smith L (2008). What benefits will choice bring to patients? Literature review and assessment of implications. J Health Serv Res Policy.

[33] Braddock Clarence H. (2012). Supporting Shared Decision Making When Clinical Evidence Is Low. Medical Care Research and Review.

[34] Zorgautoriteit N (2014). Beleidsregel TH/BR-012: Transparantie zorgaanbieders.

[35] Charles C, Gafni A, Whelan T (1999). Decision-making in the physician-patient encounter: revisiting the shared treatment decision-making model. Soc Sci Med.

[36] Meijers MC, Noordman J, Spreeuwenberg P, van Dulmen S (2019). Shared decision-making in general practice: an observational study comparing 2007 with 2015. Fam Pract.

[37] Bes R, Wendel S, de Jong J (2012). Het vertrouwensprobleem van zorgverzekeraars. ESB.

[38] Leu RE, Rutten FF, Brouwer W, Matter P, Rütschi C. The Swiss and Dutch health insurance systems: universal coverage and regulated competitive insurance markets. The Commonwealth Fund. 2009;104(1–40).

[39] van der Velden LFJ, Kasteleijn A, Kenens RJ (2017). Cijfers uit de registratie van huisartsen. Peiling 2016.

