# Supplementary material for: To what degree do patients actively choose their healthcare provider at the point of referral by their GP? A video observation study
Source: BMC Fam Pract. 2019 Dec 1;20:166. doi: 10.1186/s12875-019-1060-2 (PMC6885306; doi:10.1186/s12875-019-1060-2)
Supplement: Supplementary file 1 — Additional file 1. Observation protocol. [file 12875_2019_1060_MOESM1_ESM.docx]

| GP code: Patient code: Observation date:  Consultation duration: Name observer: |
| --- |

**Already known: complaint (ICPC-code), age, region (e.g. region we cannot make any comparisons, because most consultations took place around Nijmegen)**

**The referral in general:**

1) Is the patient being referred to a care provider or is he being prescribed medicines/aids? (multiple answers possible)

□ No referral/prescription

□ Specialist (referral required)

□ Mental care (referral required)

□ Practice supporter general practitioner mental healthcare/ 1^st^-line psychologist (no referral required)

□ Paramedic (no referral required)

□ Medication

□ A tool

□ Lab/x-ray

□ Other, namely………………………

2) Where exactly is the patient referred to/which medication/tool is prescribed?

……………………………

3) What is the reason for referral/prescribing prescription? (multiple answers possible)

□ Diagnosis

□ Treatment

□ Second opinion

**The role of the patient:**

4) How much input is there from the patient around the choice of a healthcare provider?

□ 1) little or no input □ 2) some input □ 3) a large amount of input

1 = little or no input. The GP chooses the provider and the patient simply agrees with the proposed institution or caregiver. It is obvious that the patient follows up the advice of the GP.

2 = some input. The patient is given a choice by the GP between a few providers or tells the GP that he or she does not want to be referred to a specific provider.

3 = a large amount of input. Not the GP, but the patient him or herself chooses the provider he or she is referred to or asked for alternative options. Alternatively, no decision is made during the consultation and the patient has to choose a care provider after the consultation.

5) Does the patient prefer a particular healthcare provider? (multiple answers possible)
□ Yes

a) the patient comes there regularly

b) the patient has been there before

c) the patient is working there

d) the patient has obtained this idea through family/friends/acquaintances

e) the patient has immersed himself in his/her options through media use

f) but unclear where the patient has the idea from

g) it is the nearest institution/specialist

h) the patient knows the specialist

i) patient organization advice

j) insurers’ advice

k) reimbursed by the insurer

l) Other, namely: ……………………………

□ No

6) When does the patient pronounce his preference?

□ after the doctor has given information about the option(s)

□ the moment the doctor asks the patient without having already mentioned any option(s)

□ without the doctor asking the patient or already mentioning any option(s)

7) Who mentions the option(s) in terms of a healthcare provider as first?

□ General practitioner

□ Patient

□ Is not pronounced

**The role of the general practitioner:**

8) Does the general practitioner ask for the patient’s preference for a healthcare provider?

□ Yes

□ No

9) Does the general practitioner discuss multiple options of healthcare providers?

□ Yes

□ No

10) Does the general practitioner have a preference for a particular healthcare provider?

□ Yes □ at his own initiative □ on request of the patient

□ No

11) Why does the general practitioner choose for a particular healthcare provider? (multiple answers possible)

□ Good quality, for example, based on experience of the general practitioner

□ Good quality, for example, based on information from the general practitioner

□ The patient has been here before

□ Nearest for the patient

□ Reimbursed by the insurer

□ Other, namely …………..

□ Unknown

12) Does the general practitioner provide extra information (background information) about the options (in terms of a healthcare provider)? (multiple answers possible)

□ Yes

⁯ a) Practical matters (e.g. location, opening hours)

b) Waiting list

c) Quality

d) Specialisation

e) Costs

□ No

**The influence of the health insurer or insurance policy of the patient:**

13) Are these topics discussed during consultation:

The current healthcare insurance/insurer of the patient □ Yes □ No

The additional insurance of the patient □ Yes □ No

The reimbursement of a medicine □ Yes □ No

Reimbursement of a treatment by a specialist □ Yes □ No

Reimbursement of a treatment by a mental health specialist □ Yes □ No

Reimbursement of a treatment by a paramedic □ Yes □ No

Reimbursement of specific resources (such as crutches, rollator) □ Yes □ No

The deductible of the patient □ Yes □ No

Waiting list mediation by the insurer □ Yes □ No

Help from a health insurer in choosing a healthcare provider

or with comparing different healthcare providers □ Yes □ No

Other, namely…. □ Yes □ No

14) Who takes the initiative to discuss the matters concerning the health insurance of the patient?

□ General practitioner

□ Patient
